# Computations of the M-Polynomials and Degree-Based Topological Indices for Dendrimers and Polyomino Chains

**DOI:** 10.1155/2018/1709073

**Published:** 2018-11-01

**Authors:** Young Chel Kwun, Adeel Farooq, Waqas Nazeer, Zohaib Zahid, Saba Noreen, Shin Min Kang

**Affiliations:** ^1^Department of Mathematics, Dong-A University, Busan 49315, Republic of Korea; ^2^Department of Mathematics, COMSATS University Islamabad, Lahore Campus, Lahore 54000, Pakistan; ^3^Division of Science and Technology, University of Education, Lahore 54000, Pakistan; ^4^Department of Mathematics, University of Management and Technology, Lahore 54000, Pakistan; ^5^Department of Mathematics and Statistics, The University of Lahore, Lahore, Pakistan; ^6^Department of Mathematics and RINS, Gyeongsang National University, Jinju 52828, Republic of Korea; ^7^Center for General Education, China Medical University, Taichung 40402, Taiwan

## Abstract

Topological indices correlate certain physicochemical properties like boiling point, stability, and strain energy of chemical compounds. In this report, we compute M-polynomials for PAMAM dendrimers and polyomino chains. Moreover, by applying calculus, we compute nine important topological indices of under-study dendrimers and chains.

## 1. Introduction

The** polyomino chains** constitute a finite 2-connected floor plan, where each inner face (or a unit) is surrounded by a square of length one. We can say that it is a union of cells connected by edges in a planar square lattice. For the origin of dominoes, we quote [1]. The** polyomino chains** have a long history dating back to the beginning of the 20th century, but they were originally promoted by Golomb [2, 3]. Dendrimers [4] are repetitively branched molecules. The name comes from the Greek word, which translates to “trees.” Synonymous terms for dendrimers include arborols and cascade molecules. The first dendrimer was made by Fritz Vögtle in [5]. For detailed study about dendrimer structures we refer the reader to [6–9].

Many studies have shown that there is a strong intrinsic link between the chemical properties of chemical compounds and drugs (such as melting point and boiling point) and their molecular structure [10, 11]. The topological index defined on the structure of these chemical molecules can help researchers better understand the physical characteristics, chemical reactivity, and biological activity [12]. Therefore, the study of topological indices of chemical substances and chemical structures of drugs can make up for the lack of chemical experiments and provide theoretical basis for the preparation of drugs and chemical substances. In the previous two decades, a number of topological indices have been characterized and utilized for correlation analysis in pharmacology, environmental chemistry, toxicology, and theoretical chemistry [13]. Hosoya polynomial (Wiener polynomial) [14] plays a pivotal role in finding topological indices that depend on distances. From this polynomial, a long list of distance-based topological indices can be easily evaluated. A similar breakthrough was obtained recently by Klavžar et al. [15], in the context of degree-based indices. In the year 2015, authors in [15] introduced the M-polynomial, which plays similar role “to what Hosoya polynomial does” to determine many topological indices depending on the degree of end vertices [16–20].

In the present paper, we compute M-polynomials for different dendrimer structures and polyomino chains. By applying fundamental calculus, we recover nine degree-based topological indices for these dendrimers and chains.

## 2. Basic Definitions and Literature Review

In this paper, we fixed G as a connected graph,* V (G)* is the set of vertices,* E (G)* is the set of edges, and *d*_*v*_ is the degree of any vertex* v*. Most of the definitions presented in this section can be found in [17].

**Definition 1 (see [15]).**

The M-polynomial of G is defined as

(1) $$\begin{array}{c} M\left(\;G;x,y\;\right)=\underset{\delta \leq i\leq j\leq \Delta }{\sum }m_{ij}\left(\;G\;\right)x^{i}y^{j} \end{array}$$

where *δ* = min⁡{*d*_*v*_∣*v* ∈ V(G)}, Δ = max⁡{*d*_*v*_∣*v* ∈ V(G)}, and *m*_*ij*_(*G*) is the edge *vu* ∈ *E*(*G*) that is  *i* ≤ *j*.

The very first topological index was the Wiener index, defined by Wiener in 1945, when he was studying boiling point of alkane [21]. For comprehensive details about the applications of Wiener index, see [22, 23]. After that, in 1975, Milan Randić [24] introduced the first degree-based topological index, which is now known as Randić index and is defined as

(2) $$\begin{array}{c} R_{-1/2}\left(\;G\;\right)=\underset{uv\in E\left(G\right)}{\sum }\frac{1}{\sqrt{d_{u}d_{v}}}. \end{array}$$

The generalized Randić index is defined as

(3) $$\begin{array}{c} R_{\alpha }\left(\;G\;\right)=\underset{uv\in E\left(G\right)}{\sum }\frac{1}{{\left(d_{u}d_{v}\right)}^{\alpha }}; \end{array}$$

please see [25–29].

The inverse generalized Randić index is defined as

(4) $$\begin{array}{c} RR_{\alpha }\left(\;G\;\right)=\underset{uv\in E\left(G\right)}{\sum }{\left(\;d_{u}d_{v}\;\right)}^{\alpha }. \end{array}$$

It can be seen easily that the Randić index is particular case of the generalized Randić index and the inverse generalized Randić index. Other oldest degree-based topological indices are Zagreb indices. The first Zagreb index is defined as

(5) $$\begin{array}{c} M_{1}\left(\;G\;\right)=\underset{uv\in E\left(G\right)}{\sum }\left(\;d_{u}+d_{v}\;\right) \end{array}$$

and the second Zagreb index is defined as

(6) $$\begin{array}{c} M_{2}\left(\;G\;\right)=\underset{uv\in E\left(G\right)}{\sum }\left(\;d_{u}\times d_{v}\;\right). \end{array}$$

The second modified Zagreb index is defined as

(7) Mm2G=∑uv∈EG1dudv.

For detailed study about Zagreb indices, we refer the reader to [30–32]. There are many other degree-based topological indices, for example, symmetric division index:

(8) $$\begin{array}{c} SDD\left(\;G\;\right)=\underset{uv\in E\left(G\right)}{\sum }\left\{\;\frac{\min\left(d_{u},d_{v}\right)}{\max\left(d_{u},d_{v}\right)}+\frac{\max\left(d_{u},d_{v}\right)}{\min\left(d_{u},d_{v}\right)}\;\right\} \end{array}$$

harmonic index:

(9) $$\begin{array}{c} H\left(\;G\;\right)=\underset{vu\in E\left(G\right)}{\sum }\frac{2}{d_{u}+d_{v}} \end{array}$$

inverse sum index:

(10) $$\begin{array}{c} I\left(\;G\;\right)=\underset{vu\in E\left(G\right)}{\sum }\frac{d_{u}d_{v}}{d_{u}+d_{v}} \end{array}$$

augmented Zagreb index:

(11) $$\begin{array}{c} A\left(\;G\;\right)=\underset{vu\in E\left(G\right)}{\sum }{\left\{\;\frac{d_{u}d_{v}}{d_{u}+d_{v}-2}\;\right\}}^{3}. \end{array}$$

We refer to [33–45] for detailed survey about the above defined indices and applications. Tables exhibited in [15–19] relate some notable degree-based topological indices with M-polynomial with the following notations [17]:

(12) Dx=x∂(fx,y∂x,Dy=y∂(fx,y∂y,Sx=∫0xft,ytdt,Sy=∫0yfx,ttdt,Jfx,y=fx,x,Qαfx,y=xαfx,y.

## 3. Computational Results

In this section we give our computational results.

### 3.1. M-Polynomials and Degree-Based Indices for PAMAM Dendrimers

Polyamidoamine (PAMAM) dendrimers are hyperbranched polymers with unparalleled molecular uniformity, narrow molecular weight distribution, defined size and shape characteristics, and a multifunctional terminal surface. These nanoscale polymers consist of an ethylenediamine core, a repetitive branching amidoamine internal structure, and a primary amine terminal surface. Dendrimers are “grown” off a central core in an iterative manufacturing process, with each subsequent step representing a new “generation” of dendrimer. Increasing generations (molecular weight) produce larger molecular diameters, twice the number of reactive surface sites and approximately double the molecular weight of the preceding generation. PAMAM dendrimers also assume a spheroidal, globular shape at generation 4 and above (see molecular simulation below). Their functionality is readily tailored, and their uniformity, size, and highly reactive “molecular Velcro” surfaces are the functional keys to their use. Here we consider *PD*_1_, which denote PAMAM dendrimers with trifunctional core unit generated by dendrimer generations *G*_*n*_ with* n* growth stages, and *PD*_2_, the PAMAM dendrimers with different core generated by dendrimer generators *G*_*n*_ with* n* growth stages. *DS*_1_ is kinds of PAMAM dendrimers with* n* growth stages

**Theorem 2 .**

For the PAMAM dendrimers *PD*_1_, we have

(13) MPD1,x,y=3·2nxy2+32n+1−1xy3+92n+1−1x2y2+37·2n−4x2y3.

**Proof**

Let *PD*_1_ denote PAMAM dendrimers with trifunctional core unit generated by dendrimer generations *G*_*n*_ with n growth stages.

The edge set of *PD*_1_ has following four partitions:

(14) E1,2=e=uv∈EPD1 ∣ du=1,dv=2,E1,3=e=uv∈EPD1 ∣ du=1,dv=3,E2,2=e=uv∈EPD1 ∣ du=2,dv=2,E2,3=e=uv∈EPD1 ∣ du=2,dv=3.

Now

(15) E1,2=3·2n,E1,3=6·2n−3,E2,2=18·2n−9,

and

(16) E2,3=21·2n−12.MPD1;x,y=∑i≤jmijPD1xiyj=∑1≤2m12PD1xy2+∑1≤3m13PD1x1y3+∑2≤2m22PD1x2y2+∑2≤3m23PD1x2y3=∑uv∈E1,2m12PD1xy2+∑uv∈E1,3m13PD1xy3+∑uv∈E2,2m22PD1x2y2+∑uv∈E2,3m23PD1x2y3=E1,2xy2+E1,3x1y3+E2,2x2y2+E2,3x2y3=3×2nxy2+6×2n−3xy3+18×2n−9x2y2+21×2n−12x2y3=3×2nxy2+32n+1−1xy3+92n+1−1x2y2+37×2n−4x2y3.

**Theorem 3 .**

For the PAMAM dendrimers *PD*_1_, we have

1. *M*_1_(*G*) = 105 × 2^*n*+1^ − 108.
2. *M*_2_(*G*) = 111 × 2^*n*+1^ − 117.
3. ^*m*^*M*_2_(*G*) = 2^*n*+1^ + 19 × 2^*n*−1^ − 21/4.
4. *R*_*α*_(*G*) = 3 × 2^*n*+*α*^ + (3^*α*+1^ + 2^2*α*^ × 9)(2^*n*+1^ − 1) + 2^*α*^ × 3^*α*+1^(7 · 2^*n*^ − 4).
5. *R*_*α*_(*G*) = 3 × 2^*n*−*α*^ + (1/3^*α*−1^ + 9/2^2*α*^)(2^*n*+1^ − 1) + (1/(3^*α*−1^ × 2^*α*^))(7 · 2^*n*^ − 4).
6. *SSD*(*G*) = 7 × 2^*n*+3^ + 3 × 2^*n*+1^ + 47 × 2^*n*^ − 54.
7. *H*(*G*) = (7/5) × 2^*n*+4^ − 54/5
8. *I*(*G*) = (497/20) × 2^*n*+1^ − 513/20.
9. *A*(*G*) = 3 × 2^*n*+3^ + 21 × 2^*n*+2^ + 369 × 2^*n*−2^ − 753/8.

**Proof**

Let *PD*_1_ denote PAMAM dendrimers with trifunctional core unit generated by dendrimer generations *G*_*n*_ with n growth stages. Let

(17) MG;x,y=fx,y=3×2nxy2+32n+1−1xy3+92n+1−1x2y2+37×2n−4x2y3.

Then

(18) Dxfx,y=3×2nxy2+32n+1−1xy3+182n+1−1x2y2+67×2n−4x2y3.Dyfx,y=3×2n+1xy2+92n+1−1xy3+182n+1−1x2y2+97×2n−4x2y3.,DyDxfx,y=3×2n+1xy2+92n+1−1xy3+362n+1−1x2y2+187×2n−4x2y3,SxSyfx,y=3×2n−1xy2+2n+1−1xy3+942n+1−1x2y2+127×2n−4x2y3,DxαDyαfx,y=3×2n+αxy2+3α+12n+1−1xy3+22α×92n+1−1x2y2+2α×3α7×2n−4x2y3,SxαSyα(fx,y=3×2n−αxy2+13α−12n+1−1xy3+922α2n+1−1x2y2+13α−1×2α7×2n−4x2y3,SyDxfx,y=3×2n−1xy2+2n+1−1xy3+92n+1−1x2y2+27×2n−4x2y3,SxDyfx,y=3×2n−1xy2+92n+1−1xy3+92n+1−1x2y2+927×2n−4x2y3,SxJfx,y=2nx3+32n+1−1x4+357×2n−4x5,SxJDxDyfx,y=2n+1x3+4542n+1−1x4+1857×2n−4x5,Sx3Q−2JDx3Dy3fx,y=3×2n+3x+36982n+1−1x2+127×2n−4x3.

*(1) First Zagreb Index*

(19) M1G=Dx+Dyfx,yx=y=1=105×2n+1−108.

*(2) Second Zagreb Index*

(20) $$\begin{array}{c} M_{2}\left(\;G\;\right)={\left.\;D_{y}D_{x}\left(\;f\left(\;x,y\;\right)\;\right)\;\right|}_{x=y=1}=111\times 2^{n+1}-117. \end{array}$$

*(3) Modified Second Zagreb Index*

(21) Mm2G=SxSyfx,yx=y=1=2n+1+19×2n−1−214.

*(4) Generalized Randić Index*

(22) RαG=DxαDyαfx,yx=y=1=3×2n+α+3α+1+22α×92n+1−1+2α×3α+17·2n−4.

*(5) Inverse Randić Index*

(23) RRαG=SxαSyαfx,yx=y=1=3×2n−α+13α−1+922α2n+1−1+13α−1×2α7·2n−4.

*(6) Symmetric Division Index*

(24) SSDG=SyDx+SxDyfx,yx=y=1=7×2n+3+3×2n+1+47×2n−54.

*(7) Harmonic Index*

(25) HG=2SxJfx,yx=1=HG=75×2n+4−545.

*(8) Inverse Sum Index*

(26) $$\begin{array}{c} I\left(\;G\;\right)=S_{x}JD_{x}D_{y}{\left(\;f\left(\;x,y\;\right)\;\right)}_{x=1}=\frac{497}{20}\times 2^{n+1}-\frac{513}{20}. \end{array}$$

*(9) Augmented Zagreb Index*

**Theorem 4 .**

For the PAMAM dendrimers *PD*_2_, we have

(27) MPD2,x,y=2n+2xy2+42n+1−1xy3+24·2n−11x2y2+142n+1−1x2y3.

**Proof**

Let *PD*_2_ be the PAMAM dendrimers with different core generated by dendrimer generators *G*_*n*_ with* n* growth stages. Then the edge set of *PD*_2_ has following four partitions:

(28) E1,2=e=uv∈EPD2 ∣ du=1,dv=2,E1,3=e=uv∈EPD2 ∣ du=1,dv=3,E2,2=e=uv∈EPD2 ∣ du=2,dv=2,E2,3=e=uv∈EPD2 ∣ du=2,dv=3.

Now

(29) E1,2=4·2n,E1,3=8·2n−4,E2,2=24·2n−11,

and

(30) E2,3=28·2n−14.MPD2;x,y=∑i≤jmijPD2xiyj=∑1≤2m12PD2xy2+∑1≤3m13PD2x1y3+∑2≤2m22PD2x2y2+∑2≤3m23PD2x2y3=∑uv∈E1,2m12PD2xy2+∑uv∈E1,3m13PD2xy3+∑uv∈E2,2m22PD2x2y2+∑uv∈E2,3m23PD2x2y3=E1,2xy2+E1,3x1y3+E2,2x2y2+E2,3x2y3=4·2nxy2+8·2n−4xy3+24·2n−11x2y2+28·2n−14x2y3=2n+2xy2+42n+1−1xy3+24·2n−11x2y2+142n+1−1x2y3.

**Theorem 5 .**

For the PAMAM dendrimers *PD*_2_, we have

1. *M*_1_(*G*) = 5(7 × 2^*n*+3^ − 26).
2. *M*_2_(*G*) = 37 × 2^*n*+3^ − 140.
3. ^*m*^*M*_2_(*G*) = (23/3) × 2^*n*+1^ − 77/12.
4. *R*_*α*_(*G*) = 2^*n*+*α*+2^ + (4 × 3^*α*^ + 2^*α*+1^ × 3^*α*^ × 7)(2^*n*+1^ − 1) + 2^2*α*^(24 · 2^*n*^ − 11).
5. *R*_*α*_(*G*) = 2^*n*^ + (4/3^*α*^ + 14/6^*α*^)(2^*n*+1^ − 1) + (1/2^2*α*^)(24 · 2^*n*^ − 11).
6. *SSD*(*G*) = (109/3) × 2^*n*+2^ − 197/3.
7. *H*(*G*) = (7/5) × 2^*n*+6^ − 131/10.
8. *I*(*G*) = (497/15) × 2^*n*+1^ − 154/5.
9. *A*(*G*) = 475 × 2^*n*^ − 427/2.

**Theorem 6 .**

For the PAMAM dendrimers *DS*_1_, we have

(31) MDS1;x,y=4·3nxy4+103n−1x2y2+43n−1x2y4.

**Proof**

Let *DS*_1_ be kinds of PAMAM dendrimers with n growth stages.

The edge set of *DS*_1_ has the following three partitions:

(32) E1,4=e=uv∈EDS1 ∣ du=1,dv=4,E2,2=e=uv∈EDS1 ∣ du=2,dv=2,E2,4=e=uv∈EDS1 ∣ du=2,dv=4.

Now

(33) E1,4=4·3n,E2,2=10·3n−10,

and

(34) E2,4=4·3n−4.MDS1;x,y=∑i≤jmijDS1xiyj=∑1≤4m14DS1xy4+∑2≤2m22DS1x2y2+∑2≤4m24DS1x2y4=∑uv∈E1,4m14DS1xy4+∑uv∈E2,2m22DS1x2y2+∑uv∈E2,4m24DS1x2y4=E1,4xy4+E2,2x2y2+E2,4x2y4=4·3nxy4+10·3n−10x2y2+4·3n−4x2y4=4·3nxy4+103n−1x2y2+43n−1x2y4.

**Theorem 7 .**

For the PAMAM dendrimers *DS*_1_, we have

1. *M*_1_(*G*) = 4(7 × 3^*n*+1^ − 16).
2. *M*_2_(*G*) = 8(11 × 3^*n*^ − 9).
3. ^*m*^*M*_2_(*G*) = 4 × 3^*n*^ − 3.
4. *R*_*α*_(*G*) = 3^*n*^ × 4^*α*+1^ + (2^*α*+1^ × 5 + 2^3*α*+2^)(3^*n*^ − 1).
5. *R*_*α*_(*G*) = 3^*n*^/4^*α*−1^ + (5/2^2*α*−1^ + 1/2^3*α*−2^)(3^*n*^ − 1).
6. *SSD*(*G*) = 47 × 3^*n*^ − 30
7. *H*(*G*) = (119/15) × 3^*n*^ − 19/5
8. *I*(*G*) = (278/15) × 3^*n*^ − 46/3.
9. *A*(*G*) = (3280/27) × 3^*n*^ − 112.

### 3.2. M-Polynomials and Degree-Based Indices for Polyomino Chains

From the geometric point of view, a polyomino system is a finite 2-connected plane graph in which each interior cell is encircled by a regular square. In other words, it is an edge-connected union of cells in the planar square lattice. Polyomino chain is a particular polyomino system such that the joining of the centers (set ci as the center of the ith square) of its adjacent regular composes a path *c*_1_, *c*_2_, *c*_3_,…*c*_*n*_.

Let *B*_*n*_ be the set of polyomino chains with* n* squares. There are* 2n+1* edges in every *B*_*n*_ ∈ B_*n*_, where B_*n*_ is named as a linear chain and denoted by *L*_*n*_ if the subgraph of *B*_*n*_ induced by the vertices with* d(v)=3* is a molecular graph with exactly* n-2* squares. Also, *B*_*n*_ can be called a zigzag chain and labelled as *Z*_*n*_ if the subgraph of *B*_*n*_ is induced by the vertices with* d(v)>2* is *P*_*n*_.

The angularly connected, or branched, squares constitute a link of a polyomino chain. A maximal linear chain (containing the terminal squares and kinks at its end) in the polyomino chains is called a segment of polyomino chain. Let* l(S)* be the length of* S* which is calculated by the number of squares in* S*. For any segment* S* of a polyomino chain, we get *l*(*S*) ∈ {2,3, 4,…, *n*}. Furthermore, we deduce *l*_1_ = *n* and* m=1* for a linear chain *L*_*n*_ with* n* squares and *l*_*i*_ = 2 and* m=n-1* for a zigzag chain *Z*_*n*_ with* n* squares.

In what follows, we always assume that a polyomino chain consists of a sequence of segments *S*_1_, *S*_2_, *S*_3_,…*S*_*n*_ and *L*(*S*_*i*_) = *l*_*i*_, where *m* ≥ 1 and *i* ∈ {2,3, 4,…, *m*}. We derive that ∑_*i*=1_^*m*^*l*_*i*_ = *n* + *m* − 1.

**Theorem 8 .**

For a linear polyomino chain *L*_*n*_, we have *M*(*L*_*n*_; *x*, *y*) = 2*x*^2^*y*^2^ + 4*x*^2^*y*^3^ + (3*n* − 5)*x*^3^*y*^3^.

**Proof**

Let *L*_*n*_ be the polyomino chain with* n* squares where *l*_1_ = *n* and* m=1*. *L*_*n*_ is called the linear chain.

The edge set of *L*_*n*_ has the following three partitions:

(35) E2,2=e=uv∈ELn ∣ du=2,dv=2,E2,3=e=uv∈ELn ∣ du=2,dv=3,E3,3=e=uv∈ELn ∣ du=3,dv=3.

**Now**

(36) $$\begin{array}{c} \left|\;E_{\left\{2,2\right\}}\;\right|=2, \end{array}$$

and

(37) E2,3=4,

(38) MLn;x,y=∑i≤jmijLnxiyj=∑2≤2m22Lnx2y2+∑2≤3m23Lnx2y3+∑3≤3m33Lnx3y3=∑uv∈E2,2m22Lnx2y2+∑uv∈E2,3m23Lnx2y3+∑uv∈E3,3m33Lnx3y3=E2,2x2y2+E2,3x2y3+E3,3x3y3=2x2y2+4x2y3+3n−5x3y3.

**Theorem 9 .**

For a linear polyomino chain *L*_*n*_, we have the following:

1. *M*_1_(*G*) = 18*n* − 2.
2. *M*_2_(*G*) = 27*n* − 13.
3. ^*m*^*M*_2_(*G*) = (1/3)*n* − 11/18.
4. *R*_*α*_(*G*) = 2^2*α*+1^ + 2^*α*+2^ · 3^*α*^ + 3^2*α*^(3*n* − 5).
5. *R*_*α*_(*G*) = 1/2^*α*−1^ + 2^2−*α*^/3^*α*^ + (1/3^2*α*^)(3*n* − 5).
6. *SSD*(*G*) = 6*n* + 8/3.
7. *H*(*G*) = *n* + 14/15.
8. *I*(*G*) = (9/2)*n* − 7/10.
9. *A*(*G*) = (2187/64)*n* − 573/64.

**Theorem 10 .**

Let *Z*_*n*_ be zigzag polyomino chain with n squares such that *l*_*i*_ = 2 and *m* = *n* − 1. Then

(39) MZn,x,y=2x2y2+4x2y3+2m−1x2y4+2x3y4+3n−2m−5x4y4.

**Proof**

Let *Z*_*n*_ be zigzag polyomino chain with* n* squares such that *l*_*i*_ = 2 and *m* = *n* − 1. Polyomino chain consists of a sequence of segments *S*_1_, *S*_2_,…*S*_*m*_ and *l*(*S*_*i*_) = *l*_*i*_ where *m* ≥ 1 and *i* ∈ {1,2,…, *m*}.

The edge set of *Z*_*n*_ has the following five partitions:

(40) E2,2=e=uv∈EZn ∣ du=2,dv=2,E2,3=e=uv∈EZn ∣ du=2,dv=3,E2,4=e=uv∈EZn ∣ du=2,dv=4,E3,4=e=uv∈EZn ∣ du=3,dv=4,E4,4=e=uv∈EZn ∣ du=4,dv=4.

Now

(41) E2,2=2,E2,3=4,E2,4=2m−1,E3,4=2,

and

(42) E4,4=3n−2m−5.MZn;x,y=∑i≤jmijZnxiyj=∑2≤2m22Znx2y2+∑2≤3m23Znx2y3+∑2≤4m24Znx2y4+∑3≤4m34Znx3y4+∑4≤4m44Znx4y4=∑uv∈E2,2m22Znx2y2+∑uv∈E2,3m23Znx2y3+∑uv∈E2,4m24Znx2y4+∑uv∈E3,4m34Znx3y4+∑uv∈E4,4m44Znx4y4=E2,2x2y2+E2,3x2y3+E2,4x2y4+E3,4x3y4+E4,4x4y4=2x2y2+4x2y3+2m−1x2y4+2x3y4+3n−2m−5x4y4.

**Theorem 11 .**

For the Zigzag polyomino chain *Z*_*n*_ for *n* ≥ 2, we have the following:

1. *M*_1_(*G*) = 24*n* − 4*m* − 10.
2. *M*_2_(*G*) = 48*n* − 16*m* − 40.
3. ^*m*^*M*_2_(*G*) = (1/2)*n* + (1/3)*m* + 37/18
4. *R*_*α*_(*G*) = 2^2*α*+1^ + 2^*α*+2^ × 3^*α*^ + 2^3*α*+1^(*m* − 1) + 3^*α*^ × 2^2*α*+1^ + 2^4*α*^(3*n* − 2*m* − 5).
5. *R*_*α*_(*G*) = 1/2^2*α*−1^ + 1/(3^*α*^ · 2^*α*−2^) + (1/2^3*α*−1^)(*m* − 1) + 1/(2^2*α*−1^ × 3^*α*^) + (1/2^4*α*^)(3*n* − 2*m* − 5).
6. *SSD*(*G*) = 6*n* + *m* + 11/6
7. *H*(*G*) = (3/4)*n* + (1/6)*m* + 61/60.
8. *I*(*G*) = (3/5)*n* − (4/3)*m* − 92/35.
9. *A*(*G*) = (512/9)*n* − (592/27)*m* − 118688/3375.

**Theorem 12 .**

For the polyomino chain with* n* squares and of* m* segments *S*_1_ and *S*_2_ satisfying *l*_1_ = 2 and *l*_2_ = *n* − 1, *B*_*n*_^1^  (*n* ≥ 3), we have

(43) MBn1,x,y=2x2y2+5x2y3+x2y4+3n−10x3y3+3x3y4.

**Proof**

Let *B*_*n*_^1^  (*n* ≥ 3) be the polyomino chain with* n* squares and of* m* segments *S*_1_ and *S*_2_ satisfying *l*_1_ = 2 and *l*_2_ = *n* − 1. The edge set of *B*_*n*_^1^  (*n* ≥ 3) has the following five partitions:

(44) E2,2=e=uv∈EBn1 ∣ du=2,dv=2,E2,3=e=uv∈EBn1 ∣ du=2,dv=3,E2,4=e=uv∈EBn1 ∣ du=2,dv=4,E3,3=e=uv∈EBn1 ∣ du=3,dv=3,E3,4=e=uv∈EBn1 ∣ du=3,dv=4.

Now

(45) E2,2=2,E2,3=5,E2,4=1,E3,3=3n−10,

and

(46) E3,4=3.

(47) MBn1;x,y=∑i≤jmijBn1xiyj=∑2≤2m22Bn1x2y2+∑2≤3m23Bn1x2y3+∑2≤4m24Bn1x2y4+∑3≤3m33Bn1x3y3+∑3≤4m34Bn1x3y4=∑uv∈E2,2m22Bn1x2y2+∑uv∈E2,3m23Bn1x2y3+∑uv∈E2,4m24Bn1x2y4+∑uv∈E3,3m33Bn1x3y3+∑uv∈E3,4m34Bn1x3y4=E2,2x2y2+E2,3x2y3+E2,4x2y4+E3,3x3y3+E3,4x3y4=2x2y2+5x2y3+x2y4+3n−10x3y3+3x3y4.

**Theorem 13 .**

For the polyomino chain with* n* squares and of* m* segments *S*_1_ and *S*_2_ satisfying *l*_1_ = 2 and *l*_2_ = *n* − 1, *B*_*n*_^1^  (*n* ≥ 3), we have the following:

1. *M*_1_(*G*) = 18*n*.
2. *M*_2_(*G*) = 27*n* − 8.
3. ^*m*^*M*_2_(*G*) = *n*/3 + 43/72.
4. *R*_*α*_(*G*) = 2^2*α*+1^ + 6^*α*^ × 5 + 2^3*α*^ + 3^2*α*^(3*n* − 10) + 3^*α*+1^ × 4^*α*^.
5. *R*_*α*_(*G*) = 1/2^2*α*−1^ + 5/6^*α*^ + 1/2^3*α*^ + (1/3^2*α*^)(3*n* − 10) + 1/(2^2*α*^ × 3^*α*−1^).
6. *SSD*(*G*) = 6*n* + 43/12.
7. *H*(*G*) = *n* + 6/7.
8. *I*(*G*) = 27*n* − 709/10.
9. *A*(*G*) = (2187/64)*n* − 33737/4000.

**Theorem 14 .**

For polyomino chain with *n* squares and *m* segments *S*_1_, *S*_2_,…, *S*_*m*_  (*m* ≥ 3) satisfying *l*_1_ = *l*_*m*_ = 2 and *l*_2_,…, *l*_*m*−1_ ≥ 3, *B*_*n*_^2^  (*n* ≥ 4), we have

(48) MBn2,x,y=2x2y2+2mx2y3+2x2y4+3n−2m+1x3y3+22m−3x3y4.

**Proof**

Let *B*_*n*_^2^  (*n* ≥ 4) be a polyomino chain with* n* squares and* m* segments *S*_1_, *S*_2_,…, *S*_*m*_  (*m* ≥ 3) satisfying *l*_1_ = *l*_*m*_ = 2 and *l*_2_,…, *l*_*m*−1_ ≥ 3. Then the edge set of *B*_*n*_^2^  (*n* ≥ 4) has the following five partitions:

(49) E2,2=e=uv∈EBn2 ∣ du=2,dv=2,E2,3=ne=uv∈EBn2 ∣ du=2,dv=3,E2,4=e=uv∈EBn2 ∣ du=2,dv=4,E3,3=e=uv∈EBn2 ∣ du=3,dv=3,E3,4=e=uv∈EBn2 ∣ du=3,dv=4.

Now

(50) E2,2=2,E2,3=2m,E2,4=2,E3,3=3n−6m+3,

and

(51) E4,4=4m−6.

(52) MBn2;x,y=∑i≤jmijBn2xiyj=∑2≤2m22Bn2x2y2+∑2≤3m23Bn2x2y3+∑2≤4m24Bn2x2y4+∑3≤3m33Bn2x3y3+∑3≤4m34Bn2x3y4=∑uv∈E2,2m22Bn2x2y2+∑uv∈E2,3m23Bn2x2y3+∑uv∈E2,4m24Bn2x2y4∑uv∈E3,3m33Bn2x3y3+∑uv∈E3,4m34Bn2x3y4=E2,2x2y2+E2,3x2y3+E2,4x2y4+E3,3x3y3+E3,4x3y4=2x2y2+2mx2y3+2x2y4+3n−6m+3x3y3+4m−6x3y4=2x2y2+2mx2y3+2x2y4+3n−2m+1x3y3+22m−3x3y4

**Theorem 15 .**

For polyomino chain with* n* squares and* m* segments *S*_1_, *S*_2_,…, *S*_*m*_  (*m* ≥ 3) satisfying *l*_1_ = *l*_*m*_ = 2 and *l*_2_,…, *l*_*m*−1_ ≥ 3, *B*_*n*_^2^  (*n* ≥ 4), we have the following:

1. *M*_1_(*G*) = 2(9*n* + *m* − 2).
2. *M*_2_(*G*) = 3(9*n* + 2*m* − 7).
3. ^*m*^*M*_2_(*G*) = (1/3)*n* + 7/12
4. *R*_*α*_(*G*) = 3^2*α*+1^*n* + (3^*α*^ × 2^*α*+1^ − 2 × 3^2*α*+1^ + 3^*α*^ × 2^2*α*+2^)*m* + (1 + 3^*α*+1^)2^2*α*+1^ + 2^3*α*+1^ + 3^2*α*+1^.
5. *R*_*α*_(*G*) = (1/3^2*α*−1^)*n* + (1/(3^*α*^ · 2^*α*−1^) + 2/3^2*α*−1^ + 1/(2^2*α*−2^ × 3^*α*^))*m* + (1 − 1/3^*α*−1^)(1/2^2*α*−1^) + 1/2^3*α*−1^ + 1/3^2*α*−1^.
6. *SSD*(*G*) = 6*n* + (2/3)*m* + 5/2
7. *H*(*G*) = *n* − (2/35)*m* + 20/21.
8. *I*(*G*) = (9/2)*n* + (9/35)*m* − 47/42.
9. *A*(*G*) = (2187/64)*n* + (11809/4000)*m* − 134177/8000.

## 4. Conclusions

Topological indices calculated in this paper help us to guess biological activities, chemical reactivity, and physical features of under-study dendrimers and polyomino chains. For example, Randić index is useful for determining physiochemical properties of alkanes as noticed by chemist Milan Randić in 1975. He noticed the correlation between the Randić index and several physicochemical properties of alkanes like boiling point, vapor pressure, enthalpies of formation, surface area, and chromatographic retention times. Hence our results are helpful in determination of the significance of PAMAM dendrimers and polyomino chains in pharmacy and industry.

## Data Availability

All data required for this research is included within this paper.
